# Electromagnetic Model Reliably Predicts Radar Scattering Characteristics of Airborne Organisms

**DOI:** 10.1038/srep35637

**Published:** 2016-10-20

**Authors:** Djordje Mirkovic, Phillip M. Stepanian, Jeffrey F. Kelly, Phillip B. Chilson

**Affiliations:** 1 Cooperative Institute for Mesoscale Meteorological Studies, University of Oklahoma, Norman, Oklahoma 73072, United States of America; 2 Advanced Radar Research Center and School of Meteorology, University of Oklahoma, Norman, Oklahoma 73072, United States of America; 3 Oklahoma Biological Survey and Department of Biology, University of Oklahoma, Norman, Oklahoma 73019, United States of America

## Abstract

The radar scattering characteristics of aerial animals are typically obtained from controlled laboratory measurements of a freshly harvested specimen. These measurements are tedious to perform, difficult to replicate, and typically yield only a small subset of the full azimuthal, elevational, and polarimetric radio scattering data. As an alternative, biological applications of radar often assume that the radar cross sections of flying animals are isotropic, since sophisticated computer models are required to estimate the 3D scattering properties of objects having complex shapes. Using the method of moments implemented in the WIPL-D software package, we show for the first time that such electromagnetic modeling techniques (typically applied to man-made objects) can accurately predict organismal radio scattering characteristics from an anatomical model: here the Brazilian free-tailed bat (*Tadarida brasiliensis*). The simulated scattering properties of the bat agree with controlled measurements and radar observations made during a field study of bats in flight. This numerical technique can produce the full angular set of quantitative polarimetric scattering characteristics, while eliminating many practical difficulties associated with physical measurements. Such a modeling framework can be applied for bird, bat, and insect species, and will help drive a shift in radar biology from a largely qualitative and phenomenological science toward quantitative estimation of animal densities and taxonomic identification.

Observations of airborne animals with radar date back to its early use in tracking aircraft^1^. Today, radar has become one of the many tools used by researchers to study the behavior of flying animals in the airspace, resulting in many significant biological and ecological advances^2^,^3^,^4^,^5^,^6^. While the utility of radar in these applications is undeniable, there is a growing disparity between the pace of emerging technological advances in radar systems and our ability to quantitatively interpret the resulting radar measurements of wildlife. Our ability to make quantitative biological observations and qualitative inferences from radar data has been largely limited by a lack of understanding of how radio waves interact with volant animals. Laboratory studies of radio-wave interactions with animals have produced direct measurements on a variety of organisms—primarily insects—at select wavelengths, viewing angles, and polarizations^7^,^8^,^9^,^10^,^11^,^12^. While these efforts have generated a wealth of quantitative radio-scattering information, they often only report measurements at fixed viewing angles^8^ or through a limited set of azimuth or elevation angles^11^. Similarly, these measurements typically do not provide information at multiple wavelengths or polarizations, limiting their generality in weather radar applications.

The most common quantitative application of weather radar in biology has been for estimating animal densities, passage rates, or population sizes^2^,^13^. In these applications, the scattering contribution of individual animals is characterized by their radar cross-section (RCS), which is a measure of the power density of the scattered electric field relative to that which was incident on the object^14^. Values of RCS depend upon a number of parameters, including: shape, material, and size of the scatterer; wavelength of the incident radiation; incident and scattering angle of the radiation; and the polarization of the radiation with respect to the orientation of the scatterer. Radio measurements typically yield only a subset of this information, and consequently, radar biology has often employed the simplifying assumption that airborne organisms have uniform RCSs^2^,^12^,^13^,^15^,^16^,^17^. The paucity of available biological RCS data at diverse view angles and polarizations, especially for vertebrates, can primarily be attributed to the complexity of obtaining such measurements through laboratory and field observations. One imperative for expanding radar aeroecology beyond qualitative analysis and interpretation is development of standardized techniques for quantifying radio wave scattering at variable viewing angles in ways that can be extended across taxa, as well as for different radar wavelengths and polarizations.

Analytic equations describing electromagnetic radiation interactions with simple physical objects have existed since the early 1900 s^18^. However, elegant solutions for objects with even moderately more complex shapes have remained elusive. Consequently, a detailed understanding of radio-wave scatter off complex objects must be obtained through measurements or numerical simulations^14^. Although several numerical approaches have been developed over the years to calculate RCS values for a variety of objects, few have been applied to aerial animals^19^,^20^. We examined the dual-polarization radio-wave scattering properties of the Brazilian free-tailed bat (*Tadarida brasiliensis*) using laboratory measurements, field observations, and computer modeling. In comparison to single-polarization radar, dual-polarization radars provide additional information pertaining to the shape of the object being observed, and are used when identification of the scattering object or objects is important^21^. Comparisons of the model scattering calculations with controlled measurements and field observations serve to validate the model performance and demonstrate its utility in biological radar applications.

## Methods

### Overview

The radar cross section (RCS) of a complex shape can be determined by direct radio measurement of the object or by electromagnetic modeling using one of several techniques. The tradeoffs between these approaches are based on the characteristics of the scattering objects as well as the probing wavelengths of interest. Generally speaking, RCS measurements are always possible; however, the practicality of conducting measurements on certain objects can be limited. This is typically the case for objects that are prohibitively large, such as aircraft, or especially small or unwieldy, as is the case for many flying organisms. Additional complications can arise when identical measurements must be made on scatterers of very different sizes and fragility (e.g., an aphid and a goose). In these cases, electromagnetic modeling provides a possible alternative, but not without its own limitations. With this in mind, it is often necessary to weigh the costs of accuracy, practicality, and convenience when choosing a method for RCS analysis. The following presents an overview of RCS modeling and measurement - both from theoretical and practical perspectives. It is our intention to address in detail the challenges associated with RCS analysis of flying organisms, and describe the approaches that we have applied to produce the results presented herein.

### The Method of Moments (MoM) and WIPL-D Modeling Software

Strictly speaking, calculating the RCS for any object requires solving Maxwell’s equations in their general form. However, this task is essentially unachievable in an analytic form for non-basic geometric shapes. Overcoming this issue has been achieved through numerical solutions. Computational modeling in electromagnetics dates back to the 1960 s when the first algorithms for the exact numeric field calculations were published^22^,^23^. Besides these exact techniques, a group of so-called approximation techniques based on the theory of geometric and physical optics exist. In contrast to the exact techniques, approximation techniques seek to solve equations that are valid only for certain geometries, wavelength-to-dimension ratios, or other object properties. The choice of modeling technique becomes especially important for applications in which the scattering object (i.e., scatterer) has a size that is comparable to the probing wavelength. In such cases, the RCS can exhibit large variations for relatively small frequency shifts, due to resonances within the scatterer at particular wavelengths. Consequently, for dielectric scatterers in this so-called resonant regime, use of exact modeling tools is the only accurate way to resolve the RCS. Furthermore, the capability of these tools to handle complex geometries can reconcile some observed issues in reproducing all of the polarimetric variables associated with common simplified approximations, such as those involving prolate or oblate spheroids^20^.

The method applied herein is an exact solving technique known as the Method of Moments (MoM), performed using the WIPL-D Pro 3-D Electromagnetic Solver^24^,^25^. The WIPL-D software platform uses the MoM technique to analyze 3D metallic and dielectric structures by solving the electric field integral equation for a model of an object, given some definition of the incident field properties. In short, a model of the desired object is created using interconnected plates that define a physical interface between two unlike materials. The strength of this technique is its ability to represent complex geometric objects as a group of interconnected plates, or building elements, that collectively define the shape and composition of the scatterer. Boundary conditions for both the electric and magnetic fields are imposed, forming a matrix of equivalent electric and magnetic currents (known conventionally as induced surface currents) for each of the individual building elements. These currents are represented as the product of a higher order known functional basis with an unknown coefficient, for which the matrix system is solved. The combination of these coefficients with their corresponding basis functions form the total solution of the surface induced currents, and when inserted into Maxwell’s equations, produce the resulting scattered electromagnetic field. This technique and its software implementation have been validated for simple shapes such as spheres and cylinders, as well as various manmade objects including prisms and antennas^24^,^25^, but has not been applied to biological scatterers.

**Figure 1:**
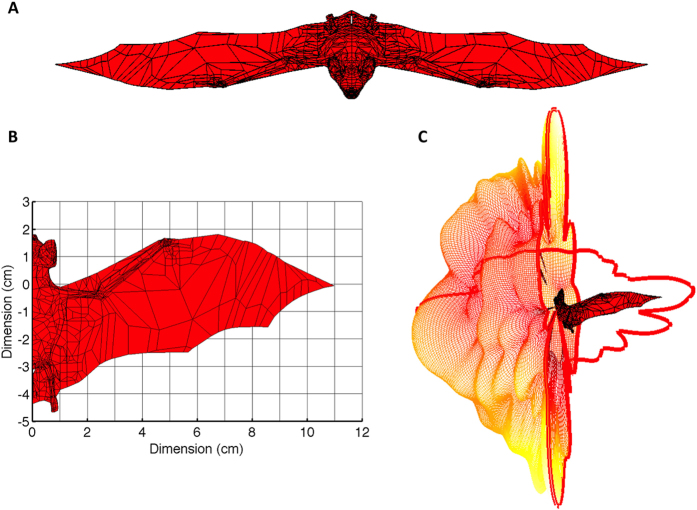
Modeling study of the radio-wave scatter from a bat. (**A**) 3-D view of the topographical surface patch model for the Brazilian free-tailed bat used in our method-of-moments calculations to find the radar cross section (RCS) of the animal assuming a probing radar wavelength of 3 cm. (**B**) Detailed view of the model from above including dimensions. (**C**) Illustration of the full solid-angle vertical-polarization RCS pattern of the bat as a function of incident angle. Values corresponding to two planar cross-sections through the bat have been highlighted for the sake of reference. All images created using Matlab R2014a (http://www.mathworks.com/).

The time requirement and computational complexity of the MoM solution is directly related to the number of coefficients that need to be calculated. The total number of these coefficients is determined by the number of topologic building elements and their electric properties^21^. With this in mind, a goal in developing an organismal model is to include only as much detail, i.e., as many plates, as is required to produce realistic scattered fields. Unfortunately, this necessary level of detail is rarely known a priori. For this study, a realistic geometric bat model was used, for which unnecessarily fine details such as toes, eyeballs, and nostrils were replaced with larger, coarser plates. The model used consists of 4404 interconnected bilinear plates (Fig. 1), representing body topology. For comparison, a more simplified second model was defined as an ellipsoid consisting of 600 plates and having similar dimensions as the combined head and torso of the bat, specifically, 50 mm along the anteroposterior axis, 15.8 mm in the dorsoventral axis, and 23.12 mm in the lateral axis.

With the geometric structure of the bat models defined, the specification of the dielectric properties enclosed or encapsulated by the building elements was required. To facilitate comparisons between the model results and laboratory measurements, the dielectric permittivity values were set to those emulating our specimen bat. This specimen was Brazilian free-tailed bat, which was collected in accordance with relevant guidelines of the University of Oklahoma’s Animal Care and Use Committee (IACUC). This protocol was approved under IACUC permit number R09-29. The specimen was collected in Harper County, Oklahoma, frozen for temporary storage, and thawed prior to measurements. With this in mind, our model was developed to match RCS signatures of the dead animal, which are expected to be slightly different than the live animal RCS^26^. As a result of this process, some of the water content of the animal had been reduced, especially in the thin membranes of the wings and tail. To account for this difference, it was possible to assign different dielectric properties to each portion of the bat’s body in the model (e.g., wings, torso, legs). However, to avoid adding complexity to the model by creating internal boundaries we opted to address this variation by using a so-called effective dielectric permittivity that represents a body-averaged value accounting for these differences. This technique yields an anatomical bat model with homogeneous permitivity throughout; that is, wings are modelled as a 1-mm dielectric skin with the same equivalent permitivity as the rest of the body. To find an appropriate effective dielectric constant, we used the Internet Electromagnetic Tissue Properties Database^27^ to find wet and dry human skin tissue dielectric values. Incidentally, the average of these skin values was approximately equal to the average of all other available tissue permittivity values, suggesting that they represent a broadly applicable biological surface permittivity. With this in mind, we assigned the complex permittivity value of *ε*_*r*_ = 29.29 − *j*12.89 to all building elements. The 4404 building elements making up the topological bat model result in 25834 unknown coefficients that define the surface current solution. To further reduce the computational complexity the option of symmetry was employed, reducing the number of unknowns by half. As a result of this symmetry, the full-body solution can be deduced by the vector sum of the original and mirrored excitations. The result of solving for these unknowns is the solution of the induced currents across the surface of the bat, which can be used to solve for the complex scattering matrix, and therefore RCS, through Maxwell’s equations.

### Controlled Radar Cross Section Measurement

To assess the validity of the RCS results obtained through the WIPL-D method of moments, direct measurements of the dead Brazilian free-tailed bat specimen discussed above were collected. Two major challenges complicated the RCS measurements of the carcass. First, the size and dielectric composition of the bat yield relatively weak echo signals that are easily overcome by surrounding clutter or environmental interference. Second, the body of the bat is quite frail and flaccid, making exact and consistent positioning difficult. Coupled with the low RCS, many typical methods for supporting the body (e.g., foam pedestals and supports) were simply too obstructive for this application. The following describes the hardware implementation used for these measurements, and discusses our methods for overcoming these technical challenges.

**Figure 2:**
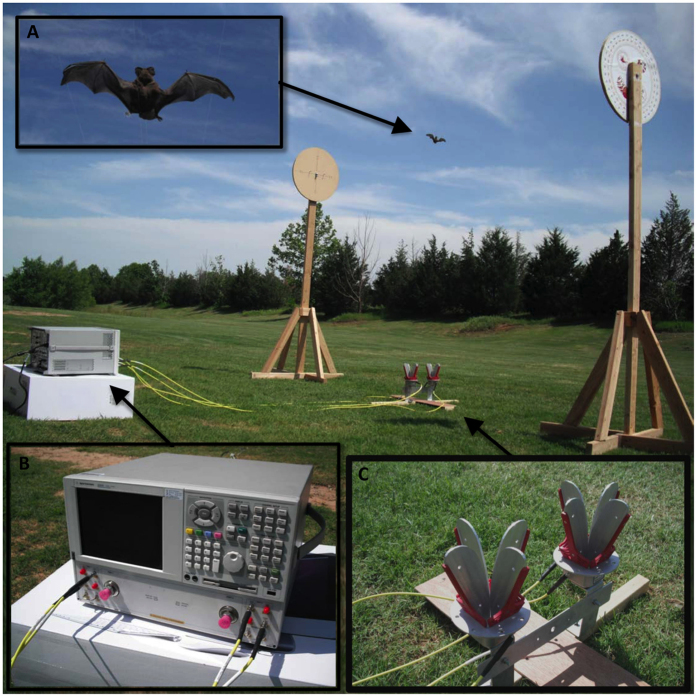
Laboratory equipment setup for controlled measurements. (**A**) Suspended Brazilian free-tailed bat specimen. (**B**) Agilent E8364B network analyzer with dual transmit and dual receive channels. (**C**) Quad-ridged dual-polarization antennas.

Since the 1980 s, network analyzers have provided the means to measure electromagnetic antenna patterns - a process technically similar to RCS measurement^28^. Over time, the capabilities of these devices have increased as their sizes decreased, resulting in a convenient tool for onsite RCS measurements. The network analyzer used in this analysis was the Agilent E8364B, a general-purpose network analyzer equipped with four receivers that may be decoupled from the instrument’s main ports using the front panel bridges. To implement direct polarimetric measurement, we decoupled the receivers and sources from the main instrument ports, and used the instrument’s sources to feed orthogonal polarizations at the transmitting antenna, while the receivers were used to sample the desired polarization return from the scatterer^28^. The instrumentation setup, presented in Fig. 2B, shows the direct connection of the source ports to the transmitting port of the quad-ridged dual-pol antennas^29^. Similarly, the receive line connects the antenna receiver to the network analyzer ports on the front panel. In this configuration, the received signal will be stored as the S-parameter matrix. To ensure reliable measurements, the transmit and receive antennas require precise control of the cross-polarization coupling and phase center position. The antennas we used are dual-polarized, ETS Lindgren quad-ridged horns (model 3164-05; Fig. 2C). Confirmed by our laboratory measurements, these antennas provide over 24 dB of isolation between orthogonal polarizations and more than 25 dB of backfire suppression^29^.

Obtaining precise and reproducible body positions when measuring such a small, limp, and fragile subject is a challenging task. It is also imperative that the apparatus suspending the bat does not interact with the measurement, either as obtrusive clutter or a source of electromagnetic coupling. Many previous measurement efforts have used polyfoam supports or enclosures, citing their negligible electromagnetic contributions^30^. While such a rig may be suitable for larger (in the RCS sense) objects, our experience has shown that the weak scattering characteristics of bats are often of the same magnitude as otherwise negligible clutter. To eliminate these clutter sources, we opted to perform outdoor sky-looking measurements and constructed a positioning apparatus that minimized interference (Fig. 2). The positioner consists of two identical wooden structures 4 m apart, with each structure being made up of a rotating disk atop a 2.3 m stationary post. A 360-degree protractor is fastened to each disk, providing rotations in 1-degree increments. Four 0.25 mm diameter low-permittivity monofilament lines span between the two counterfacing disks, piercing the bat in several places and suspending it in the middle of the rig. Upon applying slight tension to all lines, the bat was suspended in a rigid, flight-like body position above the antennas (Fig. 2A). Such positioning allowed us to measure angular dependence of the bat’s RCS at both horizontal and vertical polarizations. Care was taken when mounting the bat on the positioner to ensure the proper distance from the antennas. Values of RCS are defined in terms of plane waves with well-defined phase characteristics illuminating the object of interest. Therefore, it was necessary to place the bat within the far field of the antenna, both in terms of diameter and the antenna spacing. Moreover, it was desired to have the bat within the alias-free or unambiguous range. The settings selected in the case of our measurements placed the limit of the alias-free range at 267 m.

Post processing of the observations allows us to remove unwanted artifacts in the data and thereby improve our RCS estimation. For example, the total returned signal during the measurement consists of reflections from the scatterer under investigation (the bat in this case) along with reflections from other unwanted background signals. The background signals can be characterized by conducting measurements in the absence of the object being investigated. In this way the noise floor can be set by the averaged value of the time dependent signals from the background (here, −103dBm). Moreover, to mitigate statistical errors in amplitude measurements and reduce noise in the samples, 10 consecutive samples of the received signal were averaged in time. The temporal averaging was implemented when characterizing both the background and the scattering properties of the bat. Signal post-procession using time gating was implemented to further improve the quality of the RCS measurements by better suppressing the multipath reflections, especially those from the ground. To further clarify we have set a time filter that collects signal only if it comes within the narrow (15 cm) range gate at the position of the bat. Thus, any multipath signal that may reflect off the ground would fall outside of this time/range gate and affect the measurement.

Various techniques for RCS measurement calibrations exist but all rely on measurements of or calculations for objects having well-determined RCS characteristics, such as spheres, cylinders or plates composed of conducting material. Spheres are probably the most often used as a calibration standard. Here we used a conductive sphere with a diameter of 15.82 mm. The size of the sphere was chosen to be comparable with that of the bat in order to mitigate potential miscalibration for the case of low RCS due to the instrument’s dynamic range nonlinearity. The true (calibration) RCS for a sphere was calculated using precision computational electromagnetic tool available in WIPL-D and compared to the Mie series solution. By using two different approaches we removed any possibility of inherited errors in either of the methods. Calibration of the RCS amplitude was based on the substitution technique^14^. That is, the calibration standard (sphere) was substituted for the object being studied (bat) with measurements being conducted for each polarization separately. The same suspension system that we used to position the bat above the antennas during the RCS measurements was used for the calibration sphere. Using this configuration, the sphere could be easily located at the center-of-mass position of the bat. Moreover, since the sphere should not have cross-polarization scattering, our calibration technique was used to check for cross-polarization isolation.

### Field Observations using a Mobile Polarimetric Radar

Mobile weather radars have been widely used over the years to monitor meteorological events in areas that may be inaccessible or not adequately sampled by conventional fixed-location weather radars. Recognizing the importance of both mobile operations and the dual-polarization observations of the atmosphere, the National Severe Storms Laboratory in partnership with the University of Oklahoma developed the NOAA X-band Polarimetric (NOXP) mobile Doppler weather radar^31^. This radar simultaneously transmits and receives horizontally and vertically polarized electromagnetic waves, with a wavelength of 3.2 cm. In the summer of 2010 NOXP was deployed at several locations across Texas for the express purpose of observing Brazilian free-tailed bats. On July 10 and 11 the radar was positioned in the Texas hill country near Frio Cave (29.43N, 99.68E), which hosts a large roosting colony of Brazilian free-tailed bats. A site along a farm access road about 11.5 km SSW of the cave (29.33N, 99.71E) was selected for the operation of NOXP. The terrain was relatively flat between the radar and the cave but became hilly north of the cave. Since Brazilian free-tailed bats forage for insects during the night, NOXP was operated between 00:48–12:37 UTC on July 10 and 00:08–14:04 UTC on July 11. For that particular location and time of year, civil sunset and sunrise occur at 02:07 and 11:19 UTC, respectively.

## Results

### Model Comparisons with Controlled Measurements

**Figure 3:**
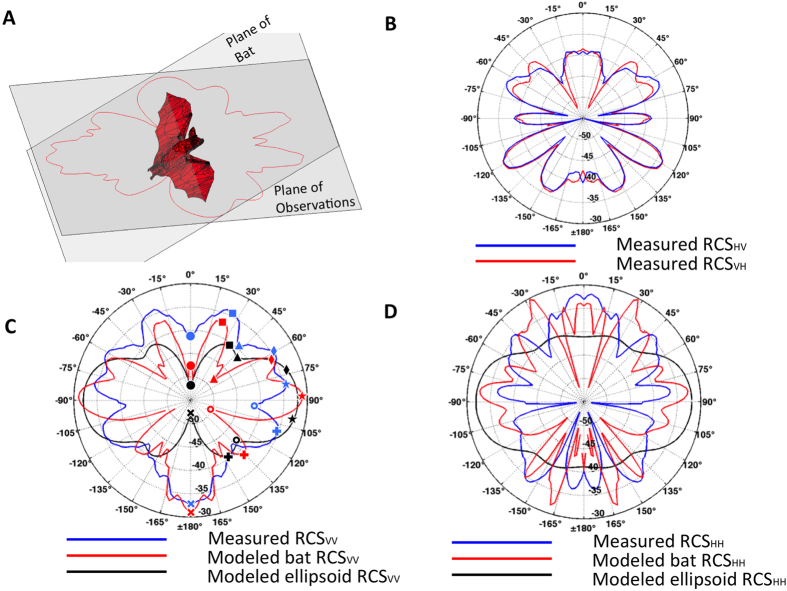
Comparison of observed and modeled polarimetric RCS components [dBsm, decibel value of the RCS relative to a reference value of 1 m^2^] of a Brazilian free-tailed bat at a 3-cm wavelength. (**A**) Orientation of the measurement plane with respect to the bat specimen. (**B**) Cross-polar RCS measurements. (**C**) Vertical polarization RCS values of the bat from measurements (blue), modeling (red), and ellipsoidal approximation (black). Comparable morphological features are annotated on each curve (see text). (**D**) Horizontal polarization RCS values of the bat from measurements (blue), modeling (red), and ellipsoidal approximation (black). All images were created using Matlab R2014a (http://www.mathworks.com/).

Following construction of the model of the bat specimen (Fig. 1A,B), the complex scattering matrix was calculated for horizontal and vertical polarizations over all solid angles of incidence (Fig. 1C). Measurements of the bat specimen yielded azimuthal polarimetric data through a single axis of rotation (Fig. 2). We precisely matched the modeled RCS results to the measurement plane of observations (35° tilt from the horizontal polarization plane) to compare the two techniques (Fig. 3A). Knowing that reciprocity of radio-wave scatter is a preserved property for linear media such as the flesh of the bat, cross-polarization scattering coefficients are expected to be identical, i.e., RCS_*HV*_ = RCS_*VH*_. Such symmetries can be used when interpreting observed and modeled RCS data, and any discrepancies relate to measurement error or unwanted received signals. The correlation of cross-polar scattering coefficients in polarimetric measurements is an especially sensitive parameter and thus provides a reliable criterion to assess measurement integrity. For this experiment, the cross-polar RCS measurements match (Fig. 3B), indicating quality measurements that can serve as the true bat scattering characteristics for comparison.

Measured and calculated RCS polar diagrams, expressed in dBsm, are provided for vertical (Fig. 3C) and horizontal (Fig. 3D) polarizations. In the figure the bat’s head/chest and rump/back correspond to azimuth angles of 0° and 180°, respectively. Qualitative comparisons can be made by identifying morphological features in the polar RCS diagrams. For example, eight morphological features have been annotated on the diagram of vertical polarization RCS for the measurement and both models (Fig. 3C). These features are local RCS maxima (i.e., lobes) or minima (i.e., nulls) and can aid visual comparisons of the diagrams. Some of these features are readily apparent in all three RCS_*VV*_ curves, e.g., a null at 0° (Fig. 3C, bullet) followed by a lobe near 30° (Fig. 3C, square), ending at a null near 45° (Fig. 3C, triangle). Other RCS_*VV*_ features that were observed in the measurements are only replicated by the anatomical bat model. For example, both the measurements and the anatomical model show two distinct lobes corresponding with the side-on aspect angle (Fig. 3C, diamond and star), while the ellipsoidal model misses this subtlety and instead produces one broad lobe. In this particular case, the morphological feature may be lost in the simplified model, but the overall RCS magnitude of this side-on lobe is still similar. As the aspect angles approach the rear flank of the bat, a local minimum (Fig. 3C, circle) is followed by another local RCS maximum apparent in all three curves (Fig. 3C, plus). Continuing clockwise, the anatomical model RCS_*VV*_ curve follows the shape of the measurements toward a maximum corresponding with a view angle toward the rear of the animal, but the ellipsoid model diverges toward a minimum (Fig. 3C, times). Because the ellipsoid has mirror symmetry across the transverse plane, it is incapable of replicating the RCS at the tail of the bat. Overall, the correspondence of these morphological markers, especially between the bat measurements and detailed model, reinforce an anatomical cause for aspect-dependent variability in RCS. Furthermore, azimuthal shifts in the location of these features (e.g., as in Fig. 3C, plus) can be attributed to physical misalignments or differences between the body of the measured bat and the anatomical model—most likely, in the position or dielectric properties of the wings (see Figs 1B and 2A).

A similar morphological comparison for horizontally-polarized RCS is less straightforward, and visual features are less distinct (Fig. 3D). The overall RCS_*HH*_ magnitudes between the measured bat and anatomical model are generally similar, but the shapes do not share as many notable morphological reference points. Understanding such a discrepancy requires us to consider the scattering surfaces contributing to the overall RCS diagram^14^. The electric field vector for the horizontal polarization is incident along the bat’s body, including the wings. Therefore, it is more susceptible to differences in the body orientation, size, and permittivity than the vertical polarization. As previously mentioned, an effective dielectric constant was used for the entire body of the modeled bat. For the measured case the dielectric constant varies across the body. For example, the wings, which are mainly composed of skin, were relatively dry compared to the rest of the bat’s body, resulting in a lower permittivity and corresponding weaker reflections than predicted by the model. This effect exists in the modeled RCS for the vertical polarization as well; however, it is not as noticeable as for the horizontal polarization. The ellipsoidal model has even less agreement with the measurements, especially from head-on and tail-on viewpoints (Fig. 3D). The source of this disparity stems from the lack of outspread wings in the ellipsoidal simplification, reducing the horizontally-oriented scattering area that would be present in the shoulders of a bat (see Fig. 1A).

Quantitative assessment of the model results with respect to the measurements are difficult because point-by-point comparisons are sensitive to slight variability in alignment. As a simple example, consider a comparison between the RCS_*VV*_ measurements and those from the anatomical bat model (Fig. 3C, blue and red, respectively). The lobes indicated by a square have a difference of 3.09 dB, and the lobes indicated by a star have a 3.13 dB offset (Fig. 3C). Although this subjective analysis indicates similar performance at these two lobes, objective analysis of the 22° look-angle would indicate an offset of 3.29, while the 90^°^ view yields a 8.18 dB descrepency—effectively comparing a local model RCS maximum (Fig. 3C, red star) to a measurement RCS minimum (Fig. 3C, blue ring). The simple result is that quantitative measures can indeed verify good performance, but poor performance metrics can be due to either true poor performance or misalignment. Furthermore, because this misalignment is not constant or linear, simple shifting methods (as by a constant lag) cannot be applied to realign the features. Keeping these caveats in mind, we do still present quantitative analysis as an objective benchmark of model performance. Taking the measured RCS as truth, Pearson correlation coefficients (*r*) are calculated using the linear RCS values (m^2^) at each azimuth to quantify the ability of the models to replicate true RCS values. Over the full set of aspect angles, the vertical polarization is better replicated by both the anatomical bat model (*r* = 0.33) and the ellipsoid (*r* = 0.06). The horizontal polarization is more affected by misalignment of lobes and nulls, resulting in negative correlations from both the anatomical (*r* = −0.19) and ellipsoidal (*r* = −0.44) models. Perhaps a more meaningful measure is the mean RCS taken over sectors of view angles. The mean vertical RCSs in linear units over the full 360° of view angles are 2.28 cm^2^ for the bat measurements, 1.89 cm^2^ for the bat model, and 1.97 cm^2^ for the ellipsoid model. The means corresponding to the horizontal polarization are 1.93 cm^2^, 1.42 cm^2^, and 2.49 cm^2^, respectively. These averages can be investigated further by considering RCS averages over a 90° sector (Table 1). In this case, the mean and median are calculated across head-on (0°), side-on (90°), and tail-on (180°) view angles for vertical and horizontal polarizations (Table 1).

### Model Comparisons with Field Observations

**Figure 4:**
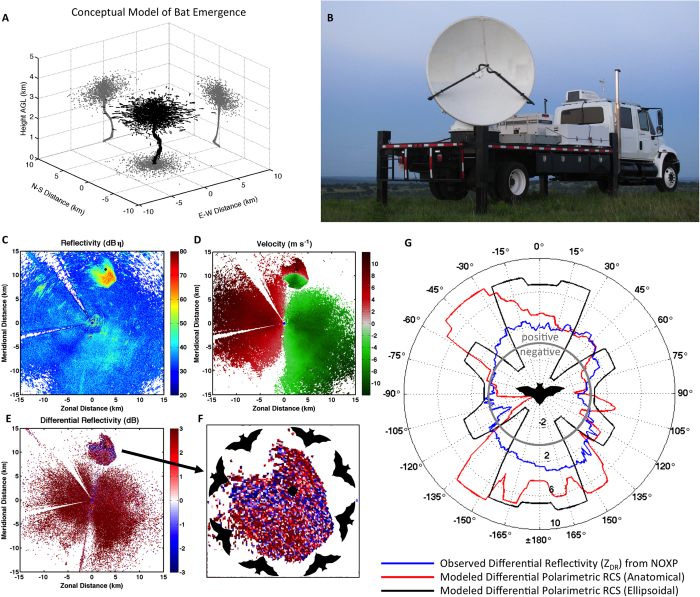
Simulated and observed bat emergence. (**A**) Conceptual representation of an emergence column and subsequent dispersal of Brazilian free-tailed bats. (**B**) Mobile radar (NOXP) deployment near Frio Cave in Texas. (**C**) Values of radar reflectivity [dB*η*, decibel value of reflectivity relative to a reference value of 1 cm^2^ km^−3^] obtained during a bat emergence from Frio Cave on 10 July 2010 at 0134 UTC (2034 local time). The cave is denoted by a black dot north-northeast of the radar (origin). (**D**) Values of radial velocity [m s^−1^] corresponding to the data shown in (**C**). Red (green) colors indicate motion away from (towards) the radar. (**E**) Values of differential reflectivity [dB] corresponding to the data shown in (**C**). (**F**) Detail of the bat emergence signature in (**E**) with schematic bat orientations overlaid. (**G**) Calculated values of differential polarimetric RCS [dB] obtained from the anatomical bat model (red), ellipsoid bat model (black), and the corresponding measurements of *Z*_*DR*_ (blue). The gray circle provides a 0-dB reference, with positive values outside and negative values within. Figure 4: Images in (**A**), (**C**), (**D**), (**E**), (**F**), and (**G**) were created using Matlab R2014a (http://www.mathworks.com/).

The Brazilian free-tailed bat is commonly detected by weather radar in the southern United States due to its abundance and the heights at which it flies (Fig. 4A), often resulting in a cloud of biological echo detected by the radar^4^ (Fig. 4C,D,E). In this case, the emergence ring signature is embedded within a background field of insects having lower reflectivity factor (Fig. 4C), widespread movement toward the northwest (Fig. 4D), and positive differential reflectivity (Fig. 4E). As individual bats exit the cave, they spread outward to minimize foraging competition (Fig. 4A). This spatial divergence results in the ability to view the bats across the full range of aspect angles from a fixed radar site (Fig. 4F). Radar measurements of reflectivity factor (*Z*, Fig. 4C) depend not only on the organism’s RCS, but also the concentration of individuals in flight. As a result, field measurements cannot validate modeled RCS; however, radar measurements of differential reflectivity (*Z*_*DR*_, Fig. 4E) are only related to the ratio of RCSs at the two polarizations, and therefore can be compared to the model. To explore the pattern in differential reflectivity, we exploited the diversity in viewing angle caused by the exodus flight away from the cave, using the NOXP radial velocity around the emergence ring to find the flight heading relative to the radar (Fig. 4F). We calculated the median *Z*_*DR*_ around the ring, which corresponds to variability in viewing angle and thus provides a field measurement of the polar variability of *Z*_*DR*_ for an ensemble of bats (Fig. 4G, blue). For comparison, we used the polar functions of RCS for each of the two models at both polarizations, corresponding to the animal’s orientation relative to the radar. Because NOXP transmits and receives both the horizontal and vertical polarizations simultaneously, the received signals contain contributions from both the co-polar wave components, as well as the cross-polar components. To make comparisons with the model results, it was necessary to include the modeled cross-polarization contributions to emulate the radar’s sampling process^20^. Because the WIPL-D produces cross-polar scattering components, this calculation simply required adding the complex co- and cross-polar scattering fields prior to computing the RCS. The resulting ratio of the RCS for the two polarizations (differential polarimetric RCS in dB) is shown in Fig. 4G. As presented by Melnikov *et al.*^20^, the effect of cross-polar contributions in the simultaneous transmission and reception configuration is an asymmetry in the polar diagrams of polarimetric variables. To further emulate the sampling effects imposed by NOXP, the modeled differential RCS is thresholded to ± 8 dB—the maximum dynamic range of NOXP. Additionally, a ± 15-degree median filter is applied to the model results to recreate the smoothing effects caused by the variable headings of the ensemble of bats in the field. Comparing the model to the field *Z*_*DR*_ observations, the trends are consistent—*Z*_*DR*_ is generally positive when when the bats are flying towards or away from the radar (i.e., viewing head-on or tail-on), and negative when the bats are observed from the side (Fig. 4G, blue). This is consistent with the pattern in differential reflectivity that we observe in the bats, which are dispersing radially from the cave (Fig. 4F). Quantitatively, the correlation between the NOXP measurements and the anatomical bat model are 0.64 around the full range of view angles, while the measurement correlation with the ellipsoid is 0.18.

### Model Dependence on Scatterer Size-to-Wavelength Ratio

**Figure 5:**
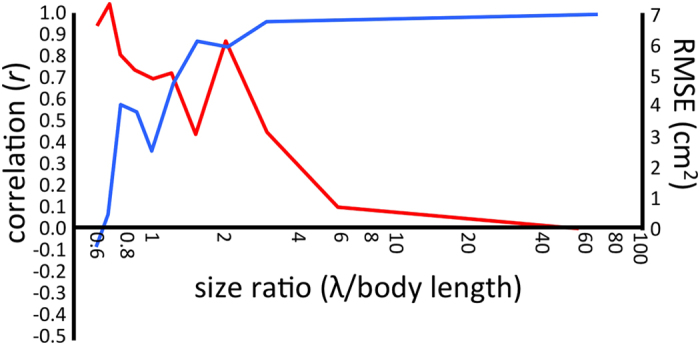
Similarity between anatomical and ellipsoidal bat models with respect to probing wavelength. Pearson correlation coefficient (blue) and root-mean-square error (red) between the modeled horizontally-polarized RCSs as a function of size ratio. The RCS values around the 360 degrees of azimuth at the 0-degree elevation view angle are all included in the calculation. A body length of 5 cm is used to calculate size ratio.

The preceding analyses have considered an X-band probing wavelength of 3 cm to facilitate comparisons with the mobile radar; however, model calculations within the WIPL-D environment can easily be set to any set or range of frequencies. At X-band, the radar wavelength is a similar scale to the physical size of the scatterer (Fig. 1B), and in these conditions the RCS is especially sensitive to changes in size or shape. We would predict that for larger wavelengths (or smaller scatterers) the sensitivity to smaller details in scatterer shape would be lessened. More specifically, we expect that as the probing wavelength increases, the details of the anatomical bat model will become less important and the RCS values will approach those of the elliptical model. To test this hypothesis, we calculate the horizontally-polarized RCS of the anatomical and elliospoidal models across a frequency sweep from 10 GHz to 100 MHz (wavelengths of 3 cm to 3 m). For each of these frequencies, the corresponding wavelength-to-bodylength ratio is calculated using the fixed body length of 5 cm, as well as the Pearson correlation coefficient and root-mean-square error (RMSE) between the anatomical and ellipsoid model RCSs at each polarization. In this case, the correlation coefficient is a measure of the morphological correspondence of the two model results, while the RMSE compares differences in RCS magnitudes (Fig. 5).

## Discussion

The electromagnetic (EM) model reliably represents the RCS of the studied bat species. As claimed in the title, a comparison of the model results to the empirical evidence of the measured RCS has shown a sufficient level of correlation to suggest that EM modeling may be capable of producing robust biota RCS. This study reveals that EM modeling can be a valuable research tool that allows higher degrees of freedom than standard RCS measurements and is easier to employ for such investigations. Additionally, EM modeling proved to provide higher number of details and better correlation to the RCS measurement than the simplified body model using a prolate ellipsoid. The correlation of the modeling and measurement results is encouraging; however, questions regarding the dielectric properties and minimum level of anatomical details are still to be explored. The study shows that the minimal level of details in the anatomical model is related to the frequency vs. dimension ratio, yet no particular study has addressed the importance of body internal structure to the RCS signature. This may be the next step in determining the necessary level of detail for modeling organismal radio scattering.

Having a detailed RCS signature of a bat specimen is necessary if radar is to be used for chiropterological studies. Even the simplest task of calculating animal densities must include the angular dependence of RCS. Yet, if observations using dual-polarization radar are to be fully exploited, more precise RCS signature data are needed to faithfully replicate radar observations by ensemble models. As observations show, bat colonies have signatures with sufficient level of details in their polarimetric variables that can be used for more accurate quantitative study of the species. Particularly, the *Z*_*DR*_ signature would not be achieved without precise anatomical modeling. Similar conclusions can be drawn for all biota at lower (lambda/dimension), as correlating ensemble modeling simulations with radar observations has proved to be a challenging, but achievable task^20^.

From this relation, a size ratio of 4 marks the approximate point where anatomical details make little difference in the magnitude and morphology of the modeled RCS patterns. That is, the 5-cm bat can be successfully approximated as an ellipsoid only when considering radar wavelengths longer than 20 cm. Similarly, when modeling scatterers smaller than one quarter of a radar wavelength, anatomical details become superfluous. In these cases, equivalent ellipsoids can be created using approximate body dimensions across the anteroposterior, dorsoventral axis, and lateral axes. This simplification can be useful for efficiently modelling a large number of taxa, especially at S-band where this size criterion is more inclusive. We must also provide a caveat that these size considerations only consider anatomical features having dielectric scattering properties, and that even large body features may be capable of being omitted if they are not composed of a scattering substance. In other words, depending on the scattering properties of the material, visually significant anatomical features may have little contribution to the electromagnetic ‘size’ of the scatterer. Notable examples may include the long tail feathers of many birds^10^ or the electromagnetically-transparent chitin of insect exoskeletons^32^, neither of which should be included when considering size ratio calculations.

The ability to advance biological radar applications, notably those dependent on polarimetry or quantification, requires complete descriptions of the radio-scattering parameters for the organisms of interest. Until now, only subsets of these data could be obtained through tedious measurements of harvested specimens, often limiting study species to those that are invasive or readily available^10^,^30^. The capability of radar for taxonomic classification of animals aloft has been widely anticipated^33^,^34^, but to date this potential has yet to be realized. The analogy in meteorological radar applications was achieved through polarimetric modeling studies of rain, snow, and hail^35^, and the resulting hydrometeor classification products have been successfully implemented in weather operations^36^. Electromagnetic modeling techniques such as those implemented in WIPL-D (www.wipl-d.com) represent the first step toward similar classification schemes for biological scatterers within the airspace.

When the identity of sampled organisms is known, radar-based quantification can be achieved using knowledge of the animal’s RCS^37^. While generalized classification is still forthcoming, some animals create single-species aggregations that can be identified on radar. These specialized cases include roosts of Brazilian Free-tailed bats^4^, Tree Swallows^38^,^39^, and Purple Martins^40^, as well as invertebrate hatches such as Mayflies^41^. In other cases, collocated observations can provide identification of bird and insect taxa to aid quantification^37^. Such techniques can provide observations of population trends over long time periods, and can help estimate the ecosystem services provided by organisms^42^; however, these capabilities are limited by the availability of RCS information for the sampled species.

We have shown for the first time that numerical electromagnetic models, such as those incorporating the method-of-moments technique, can produce robust and realistic full solid-angle radio-wave RCS data for a flying animal (here, the Brazilian free-tailed bat) at multiple polarizations. Although tested and validated for a single radar wavelength (*λ* = 3 cm) and for one type of animal, the method can easily be applied to wireframes of any species, providing data at variable wavelengths and polarizations. This is an important consideration in light of the fact that weather radars are increasingly being used to monitor the activity of volant organisms over large spatial and temporal scales^6^,^40^,^43^,^44^. Moreover, many weather radars are being upgraded to include polarimetric operation. In the United States the entire network of weather surveillance radars (NEXRAD) has already undergone such an upgrade^45^. Electromagnetic modeling of volant animals will have a dramatic impact on the field of radar aeroecology, enabling the next steps beyond qualitative characterizations into quantitative analysis.

## Additional Information

**How to cite this article**: Mirkovic, D. *et al.* Electromagnetic Model Reliably Predicts Radar Scattering Characteristics of Airborne Organisms. *Sci. Rep.* **6**, 35637; doi: 10.1038/srep35637 (2016).

## Figures and Tables

**Table 1:** Mean and median radar cross sections across 90-degree sectors for the measured bat (meas.), the anatomical bat model (anat.), and the ellipsoidal model (elli.) for vertical and horizontal polarizations.

View Angle	Meas. RCS_*VV*_	Anat. RCS_*VV*_	Elli. RCS_*VV*_	Meas. RCS_*HH*_	Anat. RCS_*HH*_	Elli. RCS_*HH*_
Mean Head-on	2.21 (−36.55)	0.80 (−41.00)	0.48 (−43.22)	2.78 (−35.56)	1.40 (−38.55)	0.97 (−40.15)
Median Head-on	1.92 (−37.17)	0.45 (−43.47)	0.46 (−43.34)	2.65 (−35.76)	0.54 (−42.70)	1.02 (−39.93)
Mean Side-on	2.37 (−36.26)	2.30 (−36.37)	3.48 (−34.58)	1.49 (−38.27)	1.27 (−38.95)	4.04 (−33.93)
Median Side-on	2.50 (−36.03)	1.51 (−38.21)	3.67 (−34.36)	1.30 (−38.85)	0.30 (−45.24)	4.38 (−33.59)
Mean Tail-on	2.20 (−36.58)	2.21 (−36.56)	0.48 (−43.22)	1.88 (−37.25)	1.76 (−37.55)	0.97 (−40.15)
Median Tail-on	1.65 (−37.83)	1.09 (−39.63)	0.46 (−43.34)	1.97 (−37.07)	1.68 (−37.74)	1.02 (−39.93)

View angles are defined such that ‘head-on’ indicates a 90-degree average over −45° to +45°, ‘side-on’ spans +45° to +135°, and ‘tail-on’ covers +135° to −135° centered on 180°. Values are given in linear units of cm^2^, follow by dBsm in parentheses.

## References

[b1] Lack D. & Varley G. C. Detection of birds by radar. Nature 156, 446–446 (1945).

[b2] Vaughn C. R. Birds and insects as radar targets: a review. Proceedings of the IEEE 73, 205–227 (1985).

[b3] Bruderer B. Naturwissenschaften 84, 45–54 (1997).

[b4] Horn J. W. & Kunz T. H. Analyzing NEXRAD Doppler radar images to assess nightly dispersal patterns and population trends in Brazilian free-tailed bats (*Tadarida brasiliensis*). Integr. Comp. Biol. 48, 24–39 (2008). 21669770 10.1093/icb/icn051

[b5] Chapman J. W., Drake V. A. & Reynolds D. R. Recent insights from radar studies of insect flight.Annu. Rev. Entomol. 56, 337–356 (2011). 21133761 10.1146/annurev-ento-120709-144820

[b6] Chilson P. B. *et al.* Partly cloudy with a chance of migration: Weather, radars, and aeroecology. B. Am. Meteorol. Soc. 93, 669–686 (2011).

[b7] Hobbs S. E. & Aldhous A. C. Insect ventral radar cross-section polarisation dependence measurements for radar entomology. IEE Proceedings - Radar, Sonar and Navigation 153, 502–508 (2006).

[b8] Hajovsky R. G., Deam A. P. & LaGrone A. H. Radar reflections from insects in the lower atmosphere. Antennas and Propagation, IEEE Transactions on 14, 224–227 (1966).

[b9] Bruderer B. Zur registrierung und interpretation von echosignaturen einem 3-cm Zielverfolgungsradar. Der Ornithologische Beobachter 66, 70–88 (1969).

[b10] Edwards J. & Houghton E. W. Radar echoing area polar diagrams of birds. Nature 184, 1059–1059 (1959).

[b11] Riley J. R. Radar cross section of insects. Proc. IEEE 73, 228–232 (1985).

[b12] Drake V. A. Estimation of unbiased insect densities and density profiles with vertically pointing entomological radars. Int. J. Remote Sens. 35, 4630–4654 (2014).

[b13] Drake V. A. Target density estimation in radar biology. Journal of Theoretical Biology 90, 545–571 (1981). 7300377 10.1016/0022-5193(81)90305-2

[b14] Knott E. F. Radar Cross Section Measurements 564 (Springer, 2012).

[b15] Martin W. J. & Shapiro A. Discrimination of bird and insect radar echoes in clear air using high-resolution radars. J. Atmos. Ocean. Tech. 24, 1215–1230 (2007).

[b16] Dokter A. M. *et al.* Bird migration flight altitudes studied by a network of operational weather radars. J. R. Soc. Interface 8, 30–43 (2011). 20519212 10.1098/rsif.2010.0116 PMC3024816

[b17] Diehl R. H., Larkin R. P. & Black J. E. Radar observation of bird migration over the Great Lakes. Auk 120, 278–290 (2003).

[b18] Mie G. Beiträge zur optik trüber medien, speziell kolloidaler metallösungen. Annalen der Physik 330, 377–445 (1908).

[b19] Schaefer G. W. Bird recognition by radar: a study in quantitative ornithology. In: Murton R. K. & Wright E. N. (ed.) The Problems of Birds as Pests. Academic Press, New York, 53–86 (1968).

[b20] Melnikov V. M., Istok M. J. & Westbrook J. K. Asymmetric echo patterns from insects. J. Atmos. Ocean. Tech. 32, 659–674 (2015).

[b21] Cloude S. R. & Pottier E. A Review of target decomposition theorems in radar polarimetry. IEEE Trans. Geosci. Remote Sens. 34, 498–518 (1996).

[b22] Harrington R. F. Field Computation by Method of Moments. (Macmillan, 1968).

[b23] Yee K. S. Numerical solution of initial boundary value problems involving Maxwell equations in isotropic media. IEEE T. Antenn. Propag., 14, 302–307 (1966).

[b24] Kolundzija B. M. & Djordjević A. R. Electromagnetic Modeling of Composite Metallic and Dielectric Structures 424 (Artech House, 2002).

[b25] Kolundzija B. M. Electromagnetic modeling of composite metallic and dielectric structures. Microwave Theory and Techniques, IEEE Transactions on 47, 1021–1032 (1999).

[b26] Drake V. A. & Reynolds D. R. Radar Entomology: Observing Insect Flight and Migration. 489 (CABI, Wallingford, UK, 2012).

[b27] Andreuccetti D., Fossi R. & Petrucci C. *IFAC-CNR, Florence (Italy), 1997. Based on data published by C. Gabriel et al. in 1996.* Available at http://niremf.ifac.cnr.it/tissprop/ (Accessed: 10th November 2015).

[b28] Agilent Technologies, New Network Analyzer Methodologies in Antenna/RCS Measurements, Available at http://cp.literature.agilent.com/litweb/pdf/5989-1937EN.pdf (Accessed: 10th November 2015).

[b29] Lindgren E. T. S. Open Bondary Quadridge Horn-Model 3164-05 Available at: http://www.ets-lindgren.com/pdf/3164-05.pdf (Accessed: 10th November 2015).

[b30] Blacksmith P. Jr. & Mack R. B. On measuring the radar cross section of ducks and chickens. Proc. IEEE 53, 1125–1125 (1965).

[b31] Schuur T., Ryzhkov A., Forsyth D., Zhang P. & Reeves H. Precipitation observations with NSSL’s X-band polarimetric radar during SNOW-V10 campaign. Pure Appl. Geophys. 171, 95–112 (2014).

[b32] Larkin R. P. & Diehl R. H.. Radar techniques for wildlife research. in Silvy N. , editor. Techniques for Wildlife Investigations and Management. Wildlife Society, Bethesda, Maryland, 319–335 (2012).

[b33] Williams T. C. & Williams J. M. A Petersons’s guide to radar ornithology? American Birds 34, 738–741 (1980).

[b34] Zrnić D. S. & Ryzhkov A. V. Observations of insects and birds with a polarimetric radar. IEEE Trans. Geosci. Remote Sens. 36, 661–668 (1998).

[b35] Straka J. M., Zrnić D. S. & Ryzhkov A. V. Bulk hydrometeor classification and quantification using polarimetric radar data: Synthesis and relations. J. Appl. Meteorol. 39, 1341–1372 (2000).

[b36] Park H., Ryzhkov A. V., Zrnić D. S. & Kim K. The hydrometeor classification algorithm for the polarimetric WSR88-D: Description and application to an MCS Weather Forecast. 24, 730–748 (2009).

[b37] Russell R. W. & Wilson J. W. Radar-observed “fine lines” in the optically clear boundary layer: Reflectivity contributions from aerial plankton and its predators. Bound-Lay. Meteorol. 82, 235–262 (1997).

[b38] Laughlin A. J., Sheldon D., Winkler D. & Taylor C. M. Behavioral drivers of communal roosting in a songbird: A combined theoretical and empirical approach. Behav. Ecol. 25, 734–743 (2014).

[b39] Laughlin A. J. *et al.* Integrating information from geolocators, weather radar, and citizen science to uncover a key stopover area of an aerial insectivore. Auk 130, 230–239 (2013).

[b40] Kelly J. *et al.* Quantigying animal phenology in the aerosphere at a continental scale using NEXRAD weather radars. Ecosphere 3, 1–9 (2012).

[b41] Masteller E. C. & Obert E. C. Excitement along the shores of lake Erie- *hexagenia* - echoes from the past. Great Lakes Research Rev. 5, 25–36 (2000).

[b42] Cleveland C. J. *et al.* Economic value of the pest control service provided by Brazilian free-tailed bats in south-central Texas. Front. Ecol. Environ. 4, 238–243 (2006).

[b43] Shamoun-Baranes J. *et al.* Continental-scale radar monitoring of aerial movement of animals. Movement Ecology 2, 9 (2014).

[b44] Dokter A. *et al.* Bird migration flight altitudes studied by a network of operational weather radars. J. R. Soc. Interface 8, 30–43 (2011). 20519212 10.1098/rsif.2010.0116 PMC3024816

[b45] Doviak R. J., Bringi V., Ryzhkov A., Zahrai A. & Zrnić D. S. Considerations for polarimetric upgrades to operational WSR-88D radars. J. Atmos. Ocean. Tech. 17, 257–278 (2000).

